# ^Fapy^dG in the Shadow of ^OXO^dG—A Theoretical Study of Clustered DNA Lesions

**DOI:** 10.3390/ijms24065361

**Published:** 2023-03-10

**Authors:** Bolesław T. Karwowski

**Affiliations:** DNA Damage Laboratory of Food Science Department, Faculty of Pharmacy, Medical University of Lodz, ul. Muszynskiego 1, 90-151 Lodz, Poland; Boleslaw.Karwowski@umed.lodz.pl

**Keywords:** 2,6-diamino-4-hydroxy-5-foramido-2′-deoxypyrimidine (^Fapy^dG), 7,8-dihydro-8-oxo-2′-deoxyguanosine (^OXO^dG), clustered DNA damage, DNA damage recognition, charge transfer, DFT

## Abstract

Genetic information, irrespective of cell type (normal or cancerous), is exposed to a range of harmful factors, which can lead to more than 80 different types of DNA damage. Of these, ^oxo^G and ^Fapy^G have been identified as the most abundant in normoxic and hypoxic conditions, respectively. This article considers d[A^Fapy^GA^OXO^GA]*[TCTCT] (oligo-^Fapy^G) with clustered DNA lesions (CDLs) containing both the above types of damage at the M06-2x/6-31++G** level of theory in the condensed phase. Furthermore, the electronic properties of oligo-^Fapy^G were analysed in both equilibrated and non-equilibrated solvation–solute interaction modes. The vertical/adiabatic ionization potential (VIP, AIP) and electron affinity (VEA, AEA) of the investigated ds-oligo were found as follows in [eV]: 5.87/5.39 and −1.41/−2.09, respectively. The optimization of the four ds-DNA spatial geometries revealed that the *trans*^Fapy^dG was energetically privileged. Additionally, CDLs were found to have little influence on the ds-oligo structure. Furthermore, for the ^Fapy^GC base-pair isolated from the discussed ds-oligo, the ionization potential and electron affinity values were higher than those assigned to ^OXO^GC. Finally, a comparison of the influence of ^Fapy^GC and ^OXO^GC on charge transfer revealed that, in contrast to the ^OXO^GC base-pair, which, as expected, acted as a radical cation/anion sink in the oligo-^Fapy^G structure, ^Fapy^GC did not significantly affect charge transfer (electron–hole and excess–electron). The results presented below indicate that 7,8-dihydro-8-oxo-2′-deoxyguanosine plays a significant role in charge transfer through ds-DNA containing CDL and indirectly has an influence on the DNA lesion recognition and repair process. In contrast, the electronic properties obtained for 2,6-diamino-4-hydroxy-5-foramido-2′deoxypyrimidine were found to be too weak to compete with ^OXO^G to influence charge transfer through the discussed ds-DNA containing CDL. Because increases in multi-damage site formation are observed during radio- or chemotherapy, understanding their role in the above processes can be crucial for the efficiency and safety of medical cancer treatment.

## 1. Introduction

Genetic information in the sequence of nucleobases of DNA is continuously exposed to harmful extra- and intra-cellular factors, both chemical and physical. These factors can lead to the formation of various types of DNA damage. To date, more than 80 different kinds of DNA lesions have been identified, including base-free sites, DNA-DNA or protein-DNA cross-links, tandem or clustered types, and sugar or base modifications [1]. From the perspective of future generations, it is highly desirable to preserve genetic information in its unchanged state. To this end, during evolution, prokaryotic and eukaryotic cells have developed DNA damage response (DDR) systems. The most common are base and nucleotide excision repair (BER, NER), mismatch repair, homologous recombination, and non-homologous end joining, all of which remove lesions in a substrate-dependent manner [2]. Disruption of these processes can lead to mutations which cause cancer and accelerate the aging process. Of all the nucleosides, 2′-deoxyguaosine is especially sensitive to harmful effects because of its particularly low oxidation potential (*E*^O^_dG/dG•_= 1.29 V) [3,4]. The lesion derived from guanosine has been identified as the most common. Its distribution depends on the endocellular environment (hypoxic or normoxic), the presence of a 2-deoxyribose substituent, and the ways in which hydroxyl radicals (●OH) are generated.

**Figure 1:**
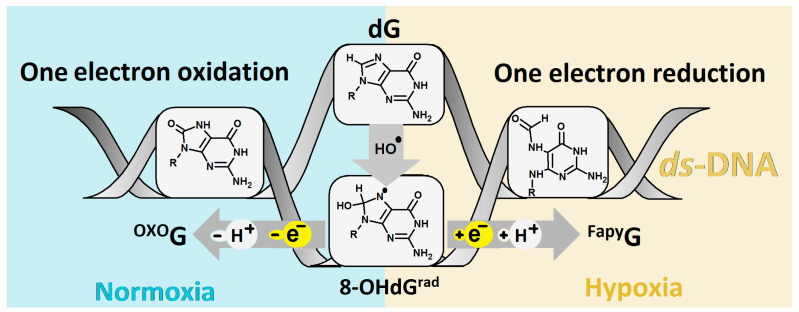
Graphical representation of ^OXO^dG and ^Fapy^dG formation in ds-DNA as a product of hydroxyl radical action, depending on cellular condition.

It should be noted that the cancer cells in close proximity to blood vases were well-oxygenated and that, as the distance increases, the condition of the microenvironment becomes hypoxic. The above oxygen gradient causes plasticity in the tumour cells and therefore increases their aggressive and metastatic phenotype. Additionally, an increase in the hypoxia-incubator factor can create an acidic environment, which results in tumour drug resistance, apoptosis/autophagy, various DNA damage, etc. For details, please refer to Shu and Simon’s recent and valuable work [5,6]. Moreover, dG can be also oxidized by other reactive oxygen and nitrogen species, free electrons, radiation, CO_3_*^●^*^–^, etc. [7,8,9,10]. The 7,8-dihydro-8-oxo-2′-deoxyguanosine (^OXO^dG) has been identified as one of the most frequent dG lesions and is keenly studied because of its relative ease of measurement, chemical synthesis, and the different methods of its introduction into the oligonucleotide structure. The second most common dG lesion is 2,6-diamino-4-hydroxy-5-foramido-2′deoxypyrimidine (^Fapy^dG) [11]. In contrast to ^OXO^dG, which is the predominant lesion formed in the presence of oxygen, ^Fapy^dG is known to be the main lesion formed in hypoxic conditions [12]. However, in both cases, the shared intermediate 2-amino-8-hydroxy-1,7,9-thihydropurine-6-one (8-OHdG^rad^), which is formed in the presence of ●OH, has been noted. Depending on the condition of the medium, 8-OHdG^rad^ can be converted into ^OXO^dG by one-electron oxidation or ^Fapy^dG by one-electron reduction, as shown in Figure 1. The above is justified by the high reaction constant of 8-OHdG^rad^ with oxygen, i.e., *k* = 2 × 10^9^ m^−1^s^−1^, while the constant of the imidazole ring-opening process has been found to be four magnitudes lower (*k* = 2 × 10^5^ m^−1^s^−1^) [13].

Dizdaroglu et al. noted the level of ^Fapy^dG in four different human breast cancer cell lines by GC/MS in a range of between 0.6 and 1.0 lesions per 10^6^ nucleosides [14]. In the same studies, ^OXO^dG was found to be between 0.07 and 0.15, depending on the cell lines. Arczewska et al. noted the frequency of ^Fapy^G in *Caenorhabditis elegans* (the medical and toxicological model organism) genome as 7.79 ± 0.47/10^6^ DNA bases, while, in the case of ^OXO^G, using the phenol extraction protocol for isolating genetic material, the frequency was 20.66 per 10^6^ [15]. Recently, Dizdaroglu et al. noted the ^OXO^G and ^Fapy^G levels as 14.62 ± 1.45 and 8.08 ± 0.9 per 10^6^ DNA bases, respectively, using the high-salt DNA extraction method [11]. As discussed above, DNA damage frequency depends on several factors, such as the detection method, cell line, and isolation protocol; however, there is no doubt that the levels of ^Fapy^G and ^OXO^G have always been at a similar level. Because of the difficulty of chemically synthesizing ^Fapy^dG into DNA, the biochemical and biological roles of this lesion are limited. However, research has indicated that the methylated form of ^Fapy^G (^ME-Fapy^G) almost completely inhibits DNA strand elongation, which suggests that it is more likely to be a lethal lesion than a mutagenic one [16]. The transversion potential G→C or G→T was noted as very low, as the polymerase inserts cytosine opposite ^ME-Fapy^G [17]. However, when ^Fapy^G was synthesized and introduced into the specified/required place of an oligonucleotide, its mutagenic potential was confirmed [18,19]. As in the case of ^OXO^G, ^Fapy^G can form a base pair (BP) with adenine; furthermore, base misincorporation was observed to occur at the same level as for oxidized guanine [20]. Therefore, the presence of 2,6-diamino-4-hydroxy-5-foramido-2′deoxypyrimidine in the genome can lead to a mutation if it is not correctly repaired. Research has shown that ^Fapy^G is effectively removed from double-stranded (ds) DNA via formamidopyrimidine-DNA glycosylase (Fpg), which initiates the cascade of BER proteins [21]. This bifunctional glycosylase cut off ^Fapy^G more effectively when it formed a canonical base-pair with cytosine instead of adenine. Other enzymes involved in base excision repair machinery can also cleave the modification under discussion. OGG1 is active towards both ^Fapy^dG and ^OXO^dG (but not ^Fapy^dA). In contrast to the above, Nei-like protein 1 (NEIL1) glycosylase removes ^Fapy^G but is inactive towards ^OXO^G [22]. Consequently, the loss of the above proteins’ activity could be the source of GC→TA transversion. To avoid such a mutation, during evolution, organisms have developed adenosine DNA glycosylase (MutY), which removes adenine from its pair with dG, ^OXO^dG, or ^Fapy^dG [23]. Even though it is not abundant, MytY [24] can scan the *Escherichia Coli* genome in less than 10 min, which would almost be impossible to achieve with the application of the classical conformation of the DNA-protein switching mechanism [25]. As an alternative to the above mechanism, DNA damage site detection through electron transfer between two proteins containing [4Fe-4S] clusters was proposed by Barton et al. [26]. Lin et al. Postulated that ^OXO^GA can accept the ejected electron and trigger MutY into action [25]. Even though this process has been investigated in the context of an ^OXO^GA base pair, the influence of ^Fapy^G on charge transfer through the double helix and its direct comparison with 7,8-dihydro-8-oxo-2′-deoxyguanosine remains unclear. With the above in mind, this article presents, for the first time, a theoretical investigation into the influence of ^Fapy^dG and ^OXO^dG as part of a clustered DNA lesion (CDL) on the excess–electron and electron–hole migration process through double-stranded DNA. In addition, because all the biological processes take place in an aqueous environment, both non-equilibrated and equilibrated solvent models were taken into theoretical consideration.

## 2. Results

**Figure 2:**
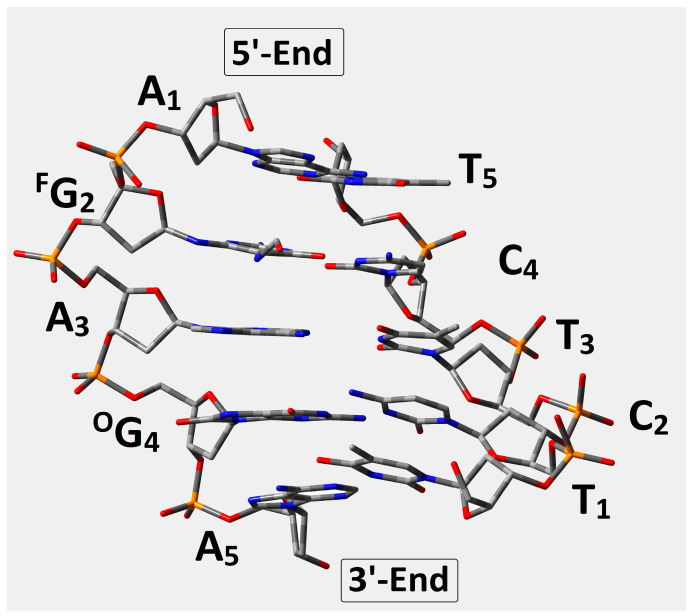
Graphical representation of d[A_1_^F^G_2_A_3_^O^G_4_A_5_]*[T_1_C_2_T_3_C_4_T_5_] optimized spatial geometry at the M06-2x/D95** level of theory in the aqueous phase using the PCM solvation model. ^F^G: 2,6-diamino-4-hydrox-5-foramido-2′deoxypyrimidine, ^O^G:7,8-dihydro-8-oxo-2′-deoxyguanosine.

**Figure 3:**
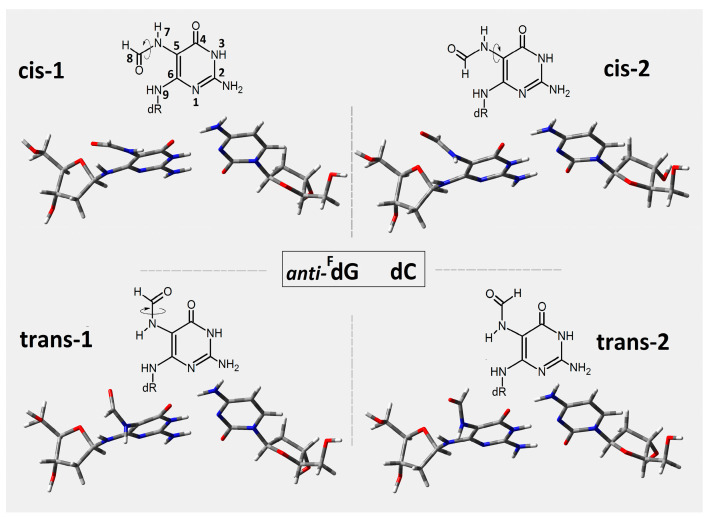
The structure of four anti-FapyG:::dC rotamers isolated from initial d[A1FG2A3OG4A5]*[T1C2T3C4T5] structure optimized on the M06-2x/D95** level of theory in aqueous phase. The cis and trans forms have been assigned according to Carell et al. [18].

In this study, the short ds-oligonucleotide containing ^Fapy^G and ^OXO^G was taken into consideration, i.e., d[A_1_**^F^G**_2_A_3_**^O^G**_4_A_5_]*[T_1_C_2_T_3_C_4_T_5_] (Figure 2), referred to throughout as *oligo-^Fapy^G*. As mentioned in the introduction, both lesions are formed via an 8-OH-dG radical intermediate initiated by •OH activity [27]. Furthermore, because the imidazole ring is open, ^Fapy^G is, in fact, the pyrimidine that bonds to the 2-deoxyribose ring via the N9 atom (Figure 3). This indicates that the ^Fapy^dG structure has a high level of conformational flexibility. Even though N9 lost its tertiary and aromatic character, ^Fapy^dG was adopted in a ds-DNA *anti*-conformation instead of *syn* [18]. Additionally, as shown in Figure 3, four rotameric forms can be selected depending on rotation around the N7-C8 and C5-N7 linkages. For the energy calculation study, the base-pair’s geometries, formed by ^Fapy^dG and dC, were isolated from the four optimized ds-pentamers containing the different rotameric forms of ^Fapy^G (Figure 3). All the ds-DNA spatial structures were fully optimized using Our Own N-layered Integrated Molecular Orbital and Molecular Mechanics (ONIOMs) method in the aqueous phase (please see the Materials and Method section). The comparison of calculated isolated base pair energies (M06-2x/6-31++G** in condensed phase) reveals the following order of stability: *trans*-2 (−1855.373892), *cis*-1 (−1855.373868), *cis*-2 (−1855.371179), *trans*-1 (−1855.367282), with all energies given in Hartree.

### 2.1. Electronic Properties and Geometry of oligo-^Fapy^G

The stability of a double helix mainly depends on the following energies: the hydrogen bond (HB) energy between base pairs, staking interaction (ST) energy, and solvation shell–solute interaction energy [28]. In the latter case, which was tangentially covered in PCM, *E*_HB_ and *E*_ST_ could be related to the HB length and the distance between the two neighbouring BPs (rise parameter), which were calculated in this study according to the standard reference frame for the description of nucleic acids [29]. As shown in Table 1, the presence of ^Fapy^G and ^OXO^G did not change the HB length and rise parameter significantly in comparison to the corresponding native ds-oligo.

In the case of the proposed ds-pentamer, both lesions are flanked by canonical base pairs from their 3′- and 5′-end sites. Additionally, ^Fapy^dG and ^OXO^dG did not interact together directly. Given this, the “global” vertical and adiabatic ionization potential and electronic affinity in non-equilibrated and equilibrated solvent interaction modes were calculated, as presented in Table 2. The results show that the complete ds-DNA and base-pair skeletons had the greatest differences in the case of vertical electron affinity calculated in the non-equilibrated mode (Δ = 0.25 eV), while, for the others, the difference was negligible. However, the appearance of ^Fapy^G and ^OXO^G in the ds-DNA structure causes a vertical and adiabatic ionization potential decrease of around 0.20eV. In contrast, almost no influence on the electron affinity properties was observed (Table 2).

The global structural changes, forced by electron attachment or electron loss, were assigned as differences between atomic positions between neutral versus anionic or cationic forms of oligo-^Fapy^G and expressed as RMSD in [Å^2^] (Table 2). As expected, the charge changes were compensated for by the flexible phosphate–sugar backbone, while the internal part containing base pairs showed almost no deviation. Furthermore, the appearance of an extra electron in the system results in an RMSD value that is about twice as high as the value for the case of a lost electron.

### 2.2. The Charge and Spin Distribution Oligo-^Fapy^G Structure

**Figure 4:**
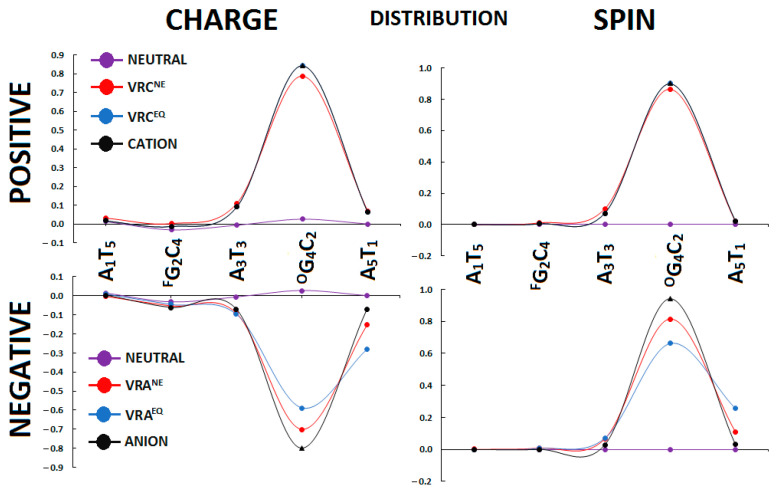
Spin and charge distribution within *oligo-^Faoy^G* calculated at the M062x/6-31++G** level of theory in the condensed phase; only the stacked base pairs were taken into consideration. VRC—vertical radical cation, VRA—vertical radical anion, NE—non-equilibrated, and EQ—equilibrated solvent–solute interaction. The charge and spin distribution of vertical and adiabatic forms. The raw data have been given in Table S2. F = Fapy, O = OXO.

It is worthwhile to investigate the influence of CDL containing two lesions formed via the same intermediate, i.e., ^Fapy^G and ^OXO^G, on charge and spin distribution. For this proposal, the Hirschfeld methodology was applied [31]. In addition, the initial stage of the charged molecule formation, after electron adoption or ejection, was examined in light of the non-equilibrated solvent–solute interaction. The radical cation or anion migrates through stacked base pairs, and, for this reason, only the internal skeleton of ds-DNA was considered in this study. As shown in Figure 4, the radical cation (electron hole) formed (charge: 84% and spin: 90%) settles mainly on the ^OXO^G_4_:::C_2_ base pair, with some dispersion over A_3_T_3_ and A_5_T_1_. The differences in charge and spin distribution between the non-equilibrated and equilibrated state of the vertical cation was less than 5% per the BPs mentioned. This finding is consistent with previous experimental and theoretical results [32,33]. It is important to note that no extra charge and spin was found within the ^Fapy^G_2_::C_4_ moiety. The situation becomes different when an extra electron is introduced into the oligo-^Fapy^G structure. The initiation point of the discussed system has been described using the non-equilibrated solvent–solute interaction. The negative charge and spin are dispersed over A_5_T_1_, ^O^G_4_C_2_, A_3_T_3_ base pairs as follows (% of charge/spin): 9/7, 70/81, and 15/11, respectively. Surprisingly, after solvent–solute equilibration, the layout discussed above changed, and the density [%] of charge/spin of the extra electron increases at A_5_T_1_ (28/26), with subsequent decreases at an ^O^G_4_C_2_(59/66) moiety, as shown in Figure 4. However, after structure relaxation towards the adiabatic radical anion forms, the negative charge was observed almost exclusively at the ^OXO^G_4_C_2_ moiety (spin 94% and charge 80%).

### 2.3. Electronic Properties of a Single Base-Pair Extracted from Oligo-^Fapy^G

For this study, the spatial geometry of oligo-^Fapy^G was optimized in its neutral, anion, and cation forms. Such a strategy allowed the calculation of each base-pair’s electronic properties, as shown in Table 3.

An analysis of the results elucidated that the ^OXO^G_4_C_2_ base-pair adopted the lowest vertical and adiabatic ionization potentials, i.e., 5.9 and 5.56 eV, respectively. For the other base pairs, the difference between VIP and AIP was negligible, which indicates that the electron–hole can travel without nucleus movement. Moreover, the vertical ionization potential value (6.17 eV) of ^Fapy^G_2_C_4_ was found comparable to that assigned for the GC base pairs extracted from corresponding unmodified ds-oligo [30]. This was partially confirmed by an analysis of the vertical and adiabatic electron affinity of the base pairs belonging to oligo-^Fapy^G. The ^OXO^GC moiety demonstrated its predisposition to excess electron adoption, with the highest absolute values of VEA (1.53 eV) and AEA (1.97 eV) being noted. As expected, the ^Fapy^GC base pairs adopted a slightly lower vertical value, i.e., 1.46 eV.

### 2.4. The Rate Constant of Charge Transfer through oligo-^Fapy^G

Charge transfer through ds-DNA can occur in its oxidized or reductive state, as described by Marcus theory, i.e., the charge transfer depends on the *k*_HT_ (rate constant), ΔG (driving force), λ (reorganization), *E*_a_ (activation), and *V*_da_ (electron coupling) energies [34]. All these parameters for this study were calculated at the M06-2x/6-31++G** level of theory in the aqueous phase. For details, please see reference [35]. Furthermore, the influence of two different DNA lesions, ^Fapy^G and ^OXO^G (forming CDL), on hole and electron migration through the double helix were taken into consideration in light of the Marcus theory. As shown in Table 4, the highest *E*_a_ was observed in the cases of electron–hole transfer between ^Fapy^G_2_C_2_ and next to A_1_T_1_ and A_3_T_3_. On the other hand, when ^Fapy^G becomes the bridge between the two BPs, i.e., A_1_T_1_ and A_3_T_3,_ *E*_a_ adopted a negative value, which results in *k*_HT_ being undetectable. The above indicates that the transfer between A_1_T_5_, A_3_T_3_, and A_5_T_1_ pairs should be unaffected and extremely fast. Additionally, the *k*_HT_ value obtained for the electron–hole transfer towards ^OXO^G was in a range of 10^8^ to 10^14^ [s^−1^]. Moreover, the influence of ^Fapy^G on the excess electron transfer was less visible in each point of deliberation, with adopted values in a range of between 10^12^ and 10^13^[s^−1^]. An exception is when ^Fapy^G becomes the bridge, i.e., A_1_T_1_←A_3_T_3_, for which the activation and reorganization become negative. This indicates that ^Fapy^G accelerated the electron transfer, as opposed to ^OXO^G, for which 4.7 × 10^−13^ [s^−1^] *k*_HT_ was found.

**Figure 5:**
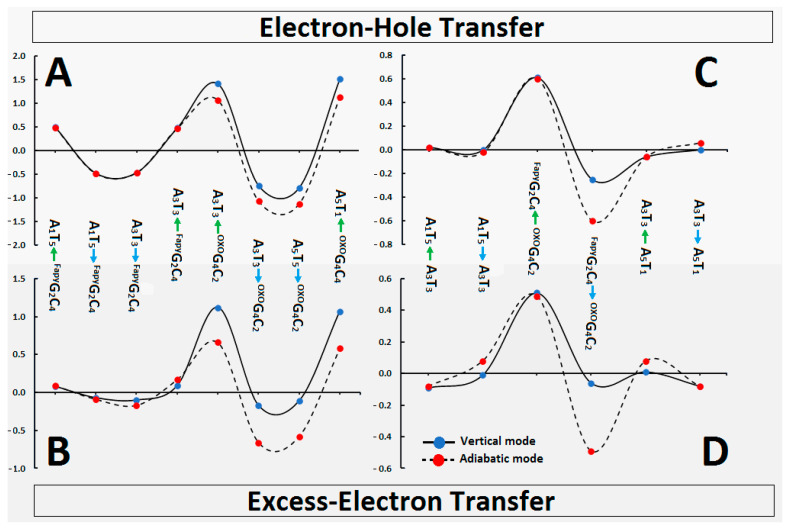
In [eV], the energy barrier profile of the charge migration process through the oligo-^Fapy^G contained CDL in vertical (black line and blue spot) and adiabatic (black dash and red spot) modes. (**A**,**B**) represented the direct transfer between neighbour base pairs, (**C**,**D**) the transfer over the single BP bridge. The numeric data has been given in Table S5.

The above results coincide well with the assigned charge energy barrier as presented in Figure 5. The charge migration barrier can be discussed in vertical and adiabatic modes on account of the nature of the process, as described in a previous study [35]. In brief, the oligo-^Fapy^G was initially divided into three ds-trimmers: A_1_^Fapy^G_2_A_3_, ^Fapy^G_2_A_3_^OXO^G_4_, and A_3_^OXO^G_4_A_5_ (the system notation was reduced to a purine strand only). The barriers of radical cation/anion transfer can be calculated as an iterative single-step super-exchange or hole/electron hopping process, as depicted in Figure 5. The details have been given in the Supplementary Materials and Table S5. As expected, the radical cation migration from the ^Fapy^GC→AT (~0.5 eV) or from the ^OXO^GC→AT (~1.4 eV) pairs were unprivileged (ΔG^Barrier^ of reaction was noted as positive; see Table S2). The above indicates that ^OXO^GC constituted a more effective electron–hole trap than ^Fapy^GC. This was confirmed by the negative ΔG^Barrier^ value of the reverse process, i.e., AT→^Fapy^GC (−0.49 eV) or AT→^OXO^GC (−1.1 eV).

The situation is similar to the previously discussed excess–electron transfer. As presented in Figure 5 and Table S2, the difference between the adiabatic and vertical modes around ^Fapy^GC was negligible. Moreover, the ΔG value ranged from 0.09 eV to −0.09 eV. As previously, the ^OXO^GC pair can be recognized as a sink of the excess electron, which was confirmed by the significantly negative ΔG values of A_3_T_3_→^OXO^G_4_C_2_, A_5_T_1_→^OXO^G_4_C_2_ and ^Fapy^G_2_C_4_→^OXO^G_4_C_2_ as follows (vertical/adiabatic mode) in [eV]: −0.17/−0.66, −0.11/−0.58, −0.06/−0.49, respectively.

## 3. Discussion

The double helix contained two main structural subunits: (A) a phosphate–sugar backbone, which is highly flexible and can compensate for geometry changes forced by external factors, and (B) a rigid internal skeleton formed by the complementary aromatic nucleo-base connected by hydrogen bonds together with a stacking interaction stabilizing the ds-oligonucleotide structure [36]. Research recognizes that the latter rigid structure allows ds-DNA to act as a nanowire capable of transferring the electron–hole (radical cation) or excess electron over a long distance, possibly more than a thousand nucleosides, according to Barton et al. [37]. This is in good agreement with research showing that glycosylases such as MutY or Endo III can communicate via ds-DNA scanning in the effective detection of lesions [38]. It should be mentioned here that other proteins involved in the replication and repair of genetic material probably use a similar mechanism, many of which contain the [4Fe-4S] cluster [39].

Of all the DNA lesions, clustered DNA lesions (CDLs), defined as two or more lesions per one or two double-helix turns, pose a particularly serious challenge for DNA repair systems [40]. During CDL removal from the genome, cell “rescue” systems try to avoid a double-strand break (DSB) formation, which is potentially lethal [41]. In the vast majority of DNA damage in the genome, ^Fapy^G was yielded by a one-electron reduction process, while ^OXO^G was yielded by one-electron oxidation. As mentioned in the introduction, these two lesions are formed preferably in conditions of hypoxia and normoxia, respectively. This suggests that they could demonstrate a different influence on charge transfer through the double helix. In the studies presented here, all the energies of d[A^Fapy^GA^OXO^GA]*[TCTCT] (oligo-^Fapy^G) with clustered DNA lesions containing both above damage types at the M06-2x/6-31++G** level of theory in the condensed phase were considered; the ds-oligo geometries in question were optimized at the M06-2x/D95**level of theory in the condensed phase. The analysis of four optimized oligo-^Fapy^G spatial geometries contained in the ^Fapy^dG structure in the two *cis* and two *trans*-rotameric forms showed the *trans*-2 to be energetically privileged (Figure 3). Therefore, the ds-oligo containing a *trans*-2 rotamer of ^Fapy^G was selected for further theoretical discussion, as depicted in Figure 2. It should be noted that, using alternative calculation methods, some research groups assigned the domination of the *cis* form over *trans* for the isolated ^Fapy^dG [18,24,42,43]. The comparative structural analysis presented in this article showed that the influence of a multi-damage site including ^Fapy^G and ^OXO^G as subunits on the discussed ds-oligo structure was noted as negligible according to the standard DNA reference frame for the analysis of structural parameters (Table 1). Moreover, the presented results are in good agreement with previous results obtained for ds-oligo containing two ^OXO^G as a clustered damage moiety [30], which indicates that the lesion only has a negligible effect on the 3D structure of ds-DNA.

The adoption or loss of an electron by ds-DNA leads to radical cation or radical anion formation, which can migrate through a double helix for more than 200Å [44]. Most studies have discussed the electronic properties of double-stranded trimers, which are a good model for discussing single lesions, but unfortunately fail in the case of CDLs [45,46]. For these reasons, investigating the influence of the CDLs on charge transfer through a double helix, as discussed in this study, seems eminently justified.

As mentioned above, the loss or adoption of an electron can initiate electron–hole or extra electron migration through ds-DNA [47]. This process is performed using charge displacement through a π-stacked base-pair ladder. The data presented in Table 2 indicates that, during vertical anion radical formation, the involvement of the sugar-phosphate backbone appears to be minimal. This observation tallies with data obtained for the corresponding native ds-oligo [30]. Additionally, the resent research of Sevilla et al. shows that, for this process, solute–solvent interaction is crucial at the beginning point [28].

These charge and spin distribution results (Figure 4) indicate that charge transfers, through a double helix containing the discussed CDL to the destination point (^OXO^G_2_C_4_), should be almost unaffected by the presence of ^Fapy^G_2_C_4_. ^OXO^G is simply preferred over ^Fapy^G as the point where the electron–hole settles in the genome. Similar to the above, the ^Fapy^G::C moiety present in the oligo-*^Fapy^*G was not affected by an extra electron, which suggests that, in the electron transfer process through the double helix, 2,6-diamino-4-hydroxy-5-foramido-2′deoxypyrimidine plays a negligible role. Moreover, 94% and 80% spin and charge, respectively, were found on ^OXO^G_4_C_2_.

The charge transfer process through ds-DNA can be discussed as single-step tunnelling, a random-walk multistep, and polaron-like hopping [48]. Each type of hole or electron migration strictly depends on the mutual positions, spatial geometries, and individual electronic properties of the base pairs. It should be pointed out here that these factors are different when they are calculated for isolated BP and BPs extracted from a ds-oligo structure, as shown in previous research [35]. An incoherent charge migration through the double helix can be observed on the hundreds of base pairs and depends on the nature of the bridge between the donor and acceptor [48]. On the other hand, at a distance of only a few BPs, tunneling paths can be noted. Even though the above mechanisms are different, the geometry and electronic properties of purine/pyrimidine moieties are crucial. ^OXO^dG, when present in the ds-oligo structure, has been identified as a radical cation sink and terminates its movement [49]. According to the results presented above, it can be assumed that charge transfer through ^Fapy^G_2_C_4_ as a bridge should be unaffected (Table 4), similar to what was noted for a canonical BP [32,50]. In addition, the ^OXO^GC moiety of clustered DNA damage can be predicted as the end point of the extra electron transfer. Furthermore, the electron transfer through the discussed ds-pentamer should not be affected other than by ^OXO^GC base pairs on account of the similarity between VEA and AEA values in their cases (Table 3). Conversely, the presence of ^Fapy^G in the investigated system effectively slows down the hole transfer via a single-step tunneling process. The above yields *k*_HT_ values close to zero even though the ΔG was negative (Table 4). Additionally, as shown in Figure 5 and Table S2, no differences between the vertical and adiabatic modes were noted for the charge transfer in the proximity of the ^Fapy^GC base-pair. Moreover, the hole-hopping barrier between A_1_T_5_←→A_3_T_3_ and A_5_T_1_←→A_3_T_3_ was measured as almost zero eV for both vertical and adiabatic modes (Figure 5). As above, the radical cation migration from ^Fapy^GC towards ^OXO^GC over the AT bridge was noted as privileged.

## 4. Materials and Methods

The starting geometries of bi-stranded oligo-^Fapy^G were built using BIOVIA Discovery Studio Visualizer v20.1.0.19295 software [51] and noted accordingly: d[A_1_^Fapy^G_2_A_3_^OXO^G_4_A_5_]*d[T_5_C_4_T_3_C_2_T_1_]. The Cartesian coordinates (i.e.: pdb files) of discussed ds-oligo structure have been given in the supplementary materials. 

The negative charges of the phosphate groups were neutralized via the addition of protons, and the other atoms were saturated using additional hydrogen atoms. The structure optimizations of oligo-^Fapy^G were performed using the Our Own N-layered Integrated Molecular Orbital and Molecular Mechanics (ONIOMs) strategy [52]. The structures of ds-oligos were divided into high—HL (nucleobases, M06-2X/D95**) and low—LL (sugar-phosphate backbone, M06-2X/sto-3G) levels of calculation [53]. All calculations were performed in the aqueous phase. The M06-2X function with an augmented and polarized valence double-ζ basis set 6-31++G** was used for energy calculations. For all the optimized geometries, charge, and spin analyses were achieved using the Hirshfeld methodology at the M06-2X/6-31++G** level of theory [31]. The electronic properties of molecules were calculated as described previously [35,54]. The transition dipole moment of excited states and the single point calculation at the M06-2X/6-31++G** level of theory were performed using time-dependent DFT (TD-DFT) methodology [55]. Electron coupling was calculated according to the Generalized Mulliken–Hush model [56]. The solvation–solute interaction was investigated in both non-equilibrium (NE) and equilibrated (EQ) modes [57]. All the calculations of the electronic properties, i.e., VIP^NE^ (vertical ionization potential in NE state), VIP^EQ^ (vertical ionization potential in EQ state), AIP (adiabatic ionization potential); VEA^NE^ (vertical electron affinity in NE state), VEA^EQ^ (vertical electron affinity in EQ state), AEA (adiabatic electron affinity), were conducted in [eV], as described previously [34]. All the above calculations were performed with the Gaussian G16 (version C.01) software package [58].

## 5. Conclusions

Genetic information, irrespective of cell type—normal or cancerous—is exposed to various harmful factors which can lead to more than 80 different types of DNA damage. Of these, ^oxo^G and ^Fapy^G have been identified as the most abundant in normoxic and hypoxic conditions, respectively.

This study considered all the energies of d[A^Fapy^GA^OXO^GA]*[TCTCT] (oligo-^Fapy^G) with clustered DNA lesions containing both above damage types at the M06-2x/6-31++G** level of theory in the condensed phase; the ds-oligo geometries in question were optimized at the M06-2x/D95** level of theory in the condensed phase. The electronic properties of oligo-^Fapy^G were analysed in equilibrated and non-equilibrated solvation–solute interaction modes.

- The vertical/adiabatic ionization potential (VIP^EQ^, AIP) and electron affinity (VEA, AEA) of the investigated ds-oligo were noted in [eV] as 5.87/5.39 and −1.41/−2.09, respectively;
- The analysis of four optimized oligo-^Fapy^G spatial geometries contained in the ^Fapy^dG structure in the two *cis* and two *trans*-rotameric forms showed the *trans*-2 to be energetically privileged. Subsequently, the influence of a multi-damage site including ^Fapy^G and ^OXO^G as subunits on the discussed ds-oligo structure was noted as negligible according to the standard DNA reference frame for the analysis of structural parameters. Furthermore, neither the adoption of an excess electron or the loss of an electron by the oligo-^Fapy^G forced a significant macromolecule structural distortion;
- Additionally, for the ^Fapy^GC base-pair isolated from the discussed ds-oligo, the ionization potential and electron affinity values were observed to be higher than those assigned for ^OXO^GC;
- These results imply that ^Fapy^GC does not have a significant effect on electron–hole or excess–electron charge transfer. In parallel to this, the ^OXO^GC base-pair becomes the radical cation/anion sink in the oligo-^Fapy^G structure, as expected.

The results presented above indicate that 7,8-dihydro-8-oxo-2′-deoxyguanosine plays a significant role in charge transfer through ds-DNA containing CDLs and indirectly has an impact on DNA lesion recognition and repair processes. Conversely, the electronic properties obtained for 2,6-diamino-4-hydroxy-5-foramido-2′deoxypyrimidine were found to be too weak to compete with ^OXO^G in influencing a charge transfer through the discussed ds-DNA containing CDLs.

The results presented in the article shed further light on clustered DNA damage, which might be useful for the safety of future generations. Man’s continual desire to colonize worlds beyond Earth requires a study of astronaut safety protocols and research into nutrition, among other things. In addition, as our longevity increases, so too do incidents of cancer. The slowdown and weakness of DNA damage repair machinery is also an age-dependent process. Therefore, it is vital to consider and further investigate “patient-friendly” radiotherapy treatments that use ionization radiation for killing cancer cells. New forays into radiotherapy are necessary to make the dose delivery more effective while reducing the detrimental and highly undesirable effects on healthy tissue surrounding a tumour. The discovery of a different kind of DNA damage raises the question of its influence, not only on the CDL repair, formation, and DNA replication processes, but also on charge transfer, and therefore on DNA damage recognition. Therefore, the results presented in this article show the possible role of ^Fapy^dG in the “DNA recognition and repair machinery” from the perspective of multiple local damaged sites composed of different lesions, i.e., ^OXO^dG.

## Figures and Tables

**Table 1:** The structural local base-pair parameter: rise and dC1′.C1′ in [Å], λ1, and λ2 in [O] according to the standard DNA reference frame of oligo-FapyG. The hydrogen bond length in [Å] (HB-1: Ade(N1), Thy(N3) and Gua(N6), Cyt(O4); HB-2: Ade(N6), Thy(O4) and Gua(N1), Cyt(N3); HB-3: Gua(N2), Cyt(O2). F = Fapy, O = OXO.

Base Pair	HB_1_	HB_2_	HB_3_	λ_1_	λ_2_	*d*_C1′.C1′_	Base Pair Dimmer	Rise
A_1_T_5_	3.03	2.82		55.4	57.9	10.4	A_1_T_5_|^F^G_2_C_4_	2.87
^F^G_2_C_4_	2.84	2.90	2.92	55.6	20.4	10.5	G_2_C_4_|A_3_T_3_	2.95
A_3_T_3_	2.97	2.88		54.6	53.1	10.5	A_3_T_3_|^O^G_2_C_4_	3.25
^O^G_2_C_4_	2.90	2.89	2.88	55.0	51.3	10.7	^O^G_2_C_4_|A_5_T_1_	3.15
A_5_T_1_	2.82	3.01		55.4	57.9	10.4		
Reference parameters calculated for native ds-oligo structure [30]								
AT	2.79	2.95		52.2	48.1	10.8	AT|GC	3.29
GC	2.87	2.87	2.88	54.4	49.6	10.7	GC|AT	2.96

**Table 2:** Electronic properties, in [eV], of oligo-^Fapy^G: Vertical (VIP), adiabatic ionization potential (AIP) and vertical (VEA), adiabatic (AEA) electron affinity calculated at the M062x/6-31++G** level of theory in the aqueous phase. ^(a,c)^ complete double helix and ^(b,d)^ base-pair skeleton, NE—non-equilibrated solvent-solute interaction, EQ—equilibrated solvent–solute interaction, * data calculated for corresponding native ds-oligo [30]. Root-Mean-Square Deviation (RMSD) of atomic positions in [Å^2^], calculated for neutral, anionic and cationic forms of oligo-^Fapy^G. The row data have been given in Table S1 of Supplementary Materials.

oligo-^Fapy^G					
VIP^NE^	VIP^EQ^	AIP	VEA^NE^	VEA^EQ^	AEA
^(a)^ 6.50	5.87	5.39	−0.90	−1.41	−2.09
^(b)^ 6.32	5.80	5.38	−0.65	−1.35	−1.94
*^(c)^ 6.72	6.08	5.65	−0.84	−1.58	−2.09
*^(d)^ 6.48	5.98	5.58	−0.60	−1.34	−1.90
**RMSD: Anion versus Neutral**			**RMSD: Cation versus Neutral**		
**ds-DNA**	**BP**	**PS-Frame**	**ds-DNA**	**BP**	**PS-Frame**
0.19	0.16	0.22	0.34	0.28	0.39
* 0.17	0.16	0.17	0.36	0.29	0.42

**Table 3:** Electronic properties of isolated base pairs from oligo-^Fapy^G: vertical (VIP), adiabatic ionization potential (AIP), and vertical (VEA) and adiabatic (AEA) electron affinity calculated at the M062x/6-31++G** level of theory in the condensed phase. The row data has been given in supplementary materials Table S3.

Electronic Properties in [eV]				
Base-Pair	VIP	AIP	VEA	AEA
A_1_T_5_	6.65	6.65	−1.39	−1.39
^Fapy^G_2_C_4_	6.17	6.16	−1.46	−1.47
A_3_T_3_	6.63	6.63	−1.38	−1.31
^OXO^G_4_C_2_	5.90	5.56	−1.53	−1.97
A_5_T_1_	6.73	6.69	−1.43	−1.39
GC**^32^**	6.13	5.83	−1.52	−1.95
AT**^32^**	6.65	6.60	−1.40	−1.40

**Table 4:** Charge transfer parameters. The ΔG-driving force, λ-reorganization energy, *E*_a_-activation energy, *V*_12_-electron coupling, and *k*_HT_-charge rate constant of permissible transfer between base pairs of *oligo-^Fapy^G*, calculated at the m062x/6-31++G** level of theory in the aqueous phase and given in eV. Arrows indicate the direction of charge migration. The row data have been given in Table S4.

Electron–Hole Transfer					
System	λ	ΔG	*E*_a_	*V*_12_	*k_HT_*(s^−1^)
A_1_T_1_→^Fapy^G_2_C_2_	0.02	−0.49	3.78	0.24	~0.0
^Fapy^G_2_C_2_←A_3_T_3_	0.01	−0.47	6.99	0.23	~0.0
A_3_T_3_→^O^G_4_C_4_	0.34	−1.07	0.40	0.40	7.53 × 10^8^
^OXO^G_4_C_4_←A_5_T_5_	0.39	−1.13	0.34	0.41	6.90 × 10^9^
A_1_T_1_→A_3_T_3_	0.00	−0.02	−0.12	0.01	ND
^Fapy^G_2_C_2_→^O^G_4_C_4_	0.35	−0.60	0.05	0.17	1.4 × 10^14^
A_3_T_5_←A_5_T_5_	0.00	−0.06	−1.70	0.05	ND
**Excess Electron Transfer**					
A_1_T_1_→^Fapy^G _2_C_2_	0.02	−0.09	0.07	0.01	1.62 × 10^12^
^Fapy^G _2_C_2_←A_3_T_3_	0.06	−0.17	0.04	0.04	2.20 × 10^13^
A_3_T_3_→^O^G _4_C_4_	0.49	−0.66	0.02	0.07	5.65 × 10^13^
^OXO^G _4_C_4_←A_5_T_5_	0.50	−0.58	0.00	0.05	5.18 × 10^13^
A_1_T_1_←A_3_T_3_	−0.06	−0.08	−0.08	0.09	ND
^Fapy^G_2_C_2_→^O^G_4_C_4_	0.44	−0.49	0.002	0.05	6.9 × 10^13^
A_3_T_5_→A_5_T_5_	0.02	−0.08	0.08	0.08	4.7 × 10^13^

## Data Availability

Not applicable.

## Supplementary Materials

The following supporting information can be downloaded at: https://www.mdpi.com/article/10.3390/ijms24065361/s1.
