# Supplementary material for: FapydG in the Shadow of OXOdG—A Theoretical Study of Clustered DNA Lesions
Source: Int J Mol Sci. 2023 Mar 10;24(6):5361. doi: 10.3390/ijms24065361 (PMC10049008; doi:10.3390/ijms24065361)
Supplement: Supplementary file 1 [file ijms-24-05361-s001.zip › ijms-2219295-supplementary.pdf]

Supplementary Materials

^{Fapy}dG in the shadow of ^{OXO}dG. A theoretical study of clustered DNA lesions.

Boleslaw Karwowski

Table S1. Hirshfeld charge and spin distribution in the shape of oligo-^{Fapy}dG (d[$A_1{}^{F}\mathbf{G}_2A_3{}^{O}\mathbf{G}_4A_5$]*[$T_1C_2T_3C_4T_5$]), only nucleosides bases were taken into consideration, calculated at the M06-2x/6-31++G** level of theory in the aqueous phase. Vertical Cation (VC^{NC}) (NE-non-equilibrated), Vertical Cation (VC^{EQ}) (EQ-equilibrated), Vertical Anion (VA^{NE}), Vertical Anion (VA^{EQ}), Adiabatic Cation (AC), Adiabatic Anion (AA) and Vertical Neutral from Cation (VNC^{NE}), Vertical Neutral from Cation (VNC^{EQ}), Vertical Neutral from Anion (VNA^{NE}), Vertical Neutral from Anion (VNA^{EQ})

oligo-^{Fapy}dG									
	Neutral	VC^{NE}		VC^{EQ}		AC		VNC^{NE}	NVC^{EQ}
	Charge	Charge	Spin	Charge	Spin	Charge	Spin	Charge	Charge
A_1T_5	0.01	0.03	0.00	0.02	0.00	0.02	0.00	0.01	0.02
G_2C_4	-0.03	0.00	0.01	-0.01	0.00	-0.01	0.00	-0.03	-0.02
A_3T_3	-0.01	0.11	0.10	0.09	0.07	0.09	0.07	0.02	0.01
G_4C_2	0.03	0.79	0.86	0.84	0.90	0.84	0.90	0.02	0.00
A_5T_1	0.00	0.07	0.02	0.06	0.02	0.06	0.02	-0.02	-0.01
oligo-^{Fapy}dG									
	VA^{NE}		VA^{EQ}		AA		VNA^{NE}	NVA^{EQ}	
	Charge	Spin	Charge	Spin	Charge	Spin	Charge	Charge	
A_1T_5	0.00	0.00	0.01	0.00	0.00	0.00	0.01	0.00	
G_2C_4	-0.05	0.01	-0.04	0.01	-0.06	0.00	-0.05	-0.06	
A_3T_3	-0.09	0.07	-0.10	0.07	-0.07	0.03	-0.02	-0.01	
G_4C_2	-0.70	0.81	-0.59	0.66	-0.80	0.94	0.04	0.06	
A_5T_1	-0.15	0.11	-0.28	0.26	-0.07	0.03	0.02	0.00	

Table S2. The energy barriers (in eV) for radical cation migration between base pairs within trimers. Vertical (**Vert**) mode, i.e. the energies of each base pair's radical cation, which were calculated for their neutral geometry. Adiabatic (**Adia**) mode i.e. the energies of each base pair's radical cation were calculated for their cation geometry. Arrows indicate the direction of Electron-hole or Excess Electron Transfer from one base pair to another e.g.: $A^+ \rightarrow G$ calculated at M06-2x/6-31++G** level of theory in the aqueous phase.

Electron-Hole Transfer									
		$A_1 \leftarrow X_2$	$A_1 \rightarrow X_2$	$X_2 \leftarrow A_3$	$X_2 \rightarrow A_3$	$A_3 \leftarrow {}^{o}G_4$	$A_3 \rightarrow {}^{o}G_4$	${}^{o}G_4 \leftarrow A_5$	${}^{o}G_4 \rightarrow A_5$
^{Fapy}G	Vert	0.50	-0.48	-0.47	0.48	1.42	-0.74	-0.79	1.52
	Adia	0.49	-0.49	-0.47	0.47	1.07	-1.07	-1.13	1.13
		$A_1 \leftarrow A_3$	$A_1 \rightarrow A_3$	$G_2 \leftarrow G_4$	$G_2 \rightarrow G_4$	$A_3 \leftarrow A_5$	$A_3 \rightarrow A_5$		
^{Fapy}G	Vert	0.02	0.00	0.61	-0.25	-0.06	0.00		
	Adia	0.02	-0.02	0.60	-0.60	-0.06	0.06		
Excess Electron Transfer									
		$A_1 \leftarrow X_2$	$A_1 \rightarrow X_2$	$X_2 \leftarrow A_3$	$X_2 \rightarrow A_3$	$A_3 \leftarrow {}^{o}G_4$	$A_3 \rightarrow {}^{o}G_4$	${}^{o}G_4 \leftarrow A_5$	${}^{o}G_4 \rightarrow A_5$
^{Fapy}G	Vert	0.08	-0.07	-0.10	0.09	1.12	-0.17	-0.11	1.07
	Adia	0.09	-0.09	-0.17	0.17	0.66	-0.66	-0.58	0.58
		$A_1 \leftarrow A_3$	$A_1 \rightarrow A_3$	$G_2 \leftarrow G^{OXO}$	$G_2 \rightarrow G_4$	$A_3 \leftarrow A_5$	$A_3 \rightarrow A_5$		
^{Fapy}G	Vert	-0.09	-0.01	0.51	-0.06	0.01	-0.08		
	Adia	-0.08	0.08	0.49	-0.49	0.08	-0.08		

Table S3. The energies (in Hartree) of Neural, Vertical Cation, Adiabatic Cation, and Vertical Neutral forms of base pairs extracted from *ds*-oligonucleotides calculated at the M06-2x/6-31 ++G** level of theory in the aqueous phase.

oligo-^{Fapy}dG	Neutral	Vertical Cation	Adiabatic Cation	Vert Neutral
A_1T_5	-921.192126	-920.947642	-920.947684	-921.19212
$^{Fapy}G_2C_4$	-1013.664403	-1013.437575	-1013.438127	-1013.664287
A_3T_3	-921.191642	-920.948008	-920.947964	-921.191909
$^{OXO}G_4C_2$	-1012.478332	-1012.261402	-1012.274011	-1012.465364
A_5T_1	-921.192121	-920.944861	-920.94634	-921.192147
**oligo-^{Fapy}dG**		**Vertical Anion**	**Adiabatic Anion**	**Vert Neutral**
A_1T_5		-921.24338	-921.243093	-921.191985
$^{Fapy}G_2C_4$		-1013.718032	-1013.718519	-1013.664344
A_3T_3		-921.24252	-921.23965	-921.189763
$^{OXO}G_4C_2$		-1012.534634	-1012.550616	-1012.458731
A_5T_1		-921.244508	-921.243196	-921.191001

Table S4. The energies (in Hartree) of Neural, Vertical Cation (VC^{NC}) (NE-non-equilibrated), Vertical Cation (VC^{EQ}) (EQ-equilibrated), Vertical Anion (VA^{NE}), Vertical Anion (VA^{EQ}), Adiabatic Cation (AC), Adiabatic Anion (AA) and Vertical Neutral from Cation (VNC^{NE}), Vertical Neutral from Cation (VNC^{EQ}), Vertical Neutral from Anion (VNA^{NE}), Vertical Neutral from Anion (VNA^{EQ}) of complete DNA double helix and base pairs skeleton extracted from *ds*-oligonucleotides calculated at the M06-2x/6-31+G** and M06-2X/6-31++G** level of theory in the aqueous phase, respectively.

	**Neutral**	**VC^{NE}**	**VC^{EQ}**	**VA^{NE}**	**VA^{EQ}**	**AC**	**AA**	**VNC^{NE}**	**VNC^{EQ}**	**VNA^{NE}**	**VNA^{EQ}**
	**Complete DNA double helix**										
**oligo-^{Fapy}dG**	-12927,97994	-12927,764135	-12927,76414	-12928,031749	-12928,03175	-12927,781776	-12928,05691	-12927,94313	-12927,96663	-12927,93372	-12927,95834
**Base Pairs skeleton extracted from *ds*-oligonucleotides**											
**oligo-^{Fapy}dG**	-4789,818113	-4789,586013	-4789,604826	-4789,842170	-4789,867776	-4789,620442	-4789,889409	-4789,792873	-4789,803322	-4789,792632	-4789,797779

Table S5a. The Energies: Ground (E^{GR}) and Excitation (E^{EX}) state energies and Excitation and HOMO Energies as well as corresponding Dipole Moments Ground, Excitation, and Transition (DM^{G}, DM^{EX}, D_{12}) in Debays of neighbor base pair extracted from selected dimmers of *ds*-oligonucleotides, calculated at the M06-2x/6-31++G** level of theory in the aqueous phase using the DFT or TD-DFT methodology.

d[A_1 $^{Fapy}G_2$ A_3 $^{O}G_4$ A_5]*[$T_1C_2T_3C_4T_5$]										
**SYSTEM**	**B.P. Dimer**	E^{GR}	DM^{GR}	E^{EX}	DM^{EX}	D_{12}	E^{HOMO}	E^{HOMO-1}	E^{LUMO}	E^{LUMO+1}
^{Fapy}G	A_1|| $^{Fapy}G_2$	-1934.877984	12.34	-1934.745981	12.59	7.31	-0.2655	-0.2831	-0.0132	-0.0122
	$^{Fapy}G_2$||A_3	-1934.880981	11.33	-1858.332663	9.12	14.98	-0.2622	-0.2792	-0.0154	-0.0125
	A_3||$^{oxo}G_4$	-1933.694406	16.26	-1933.568847	14.56	4.92	-0.2522	-0.2824	-0.0172	-0.0123
	$^{oxo}G_4$||A_5	-1933.696078	16.45	-1933.569606	14.77	4.57	-0.2531	-0.2835	-0.0179	-0.0142

Table S5b. The Energies: Ground (E^{GR}) and Excitation (E^{EX}) state energies and Excitation and HOMO Energies as well as corresponding Dipole Moments Ground, Excitation, and Transition (DM^{G}, DM^{EX}, D_{12}) in Debays of distal base pair extracted from selected trimmers of *ds*-oligonucleotides, calculated at the M06-2x/6-31++G** level of theory in the aqueous phase using the DFT or TD-DFT methodology

d[A_1 $^{Fapy}G_2$ A_3 $^{O}G_4$ A_5]*[$T_1C_2T_3C_4T_5$]										
**SYSTEM**	**Base Pair Dimer**	E^{GR}	DM^{GR}	E^{EX}	DM^{EX}	D_{12}	E^{HOMO}	E^{HOMO-1}	E^{LUMO}	E^{LUMO+1}
^{Fapy}G	A_1||A_3	-1934,877984	12,34	-1934,745981	12,59	7,31	-0,26552	-0,28305	-0,01316	-0,01216
	$^{Fapy}G_2$||G_4	-1934,745981	11,33	-1858,332663	9,12	14,98	-0,26216	-0,27919	-0,01545	-0,0125
	A_3||A_5	-1933,694406	16,26	-1933,568847	14,56	4,92	-0,25224	-0,28243	-0,01723	-0,01233
